# Bone marrow concentrate intradiscal injection for chronic discogenic low back pain: A double-blind randomized sham-controlled trial^☆^

**DOI:** 10.1016/j.inpm.2025.100611

**Published:** 2025-07-17

**Authors:** David Levi, Sara Tyszko, Scott Horn, Nicole Pham, Joshua Levin

**Affiliations:** a Jordan-Young Institute, Virginia Beach, VA, USA; b PM&R Section, Department of Orthopaedic Surgery and Neurosurgery, Stanford University, Redwood City, CA, USA

## Abstract

**Summary of background:**

The field of orthobiologics has attempted to address the challenge of discogenic low back pain (LBP). Research in areas such as stem cells, platelet-rich plasma, and specific growth factor injections has seen limited success.

**Objective:**

The purpose of this trial was to determine the efficacy of a single intradiscal bone marrow concentrate (BMC) injection on pain and function for chronic discogenic LBP.

**Methods:**

Patients with presumed discogenic LBP participated in a prospective, double-blind, randomized, sham controlled trial of a single intradiscal BMC injection compared to a sham procedure. Pain and function were assessed at baseline, 3, 6, and 12 months by Clinical Outcome Measurement Brief Instrument (COMBI) which includes the Numeric Rating Scale (NRS). Function was also assessed by the Oswestry Disability Index (ODI). The primary outcome was based upon clinical success, defined by at least 50 % pain relief from baseline to 3, 6, and 12 months.

**Results:**

Sixty-three patients were included in the trial (45 BMC, 18 sham). There were no significant differences in the primary outcome (>50 % relief in NRS) at 3 months (40 % BMC group [95 %CI: 27–50 %] vs 33 % sham group [95 %CI: 15–56 %]), 6 months (40 % BMC [95 %CI: 27–50 %] vs 39 % sham [95 %CI: 20–61 %]), or 12 months (44 % BMC [95 %CI: 31–59 %] vs 56 % sham [95 %CI: 34–75 %]). There were no significant differences in the proportion of patients achieving at least a 30 % improvement on the ODI at 3 months (51 % BMC group [95 %CI: 37–65 %] vs 33 % sham group [95 %CI: 16–56 %]), 6 months (53 % BMC [95 %CI: 39–67 %] vs 44 % sham [95 %CI: 25–65 %]), or 12 months (56 % BMC [95 %CI: 41–69 %] vs 61 % sham [95 %CI: 39–80 %]).

**Conclusions:**

Intradiscal BMC was equivalent to a sham procedure for chronic discogenic LBP. Both groups had a high, but statistically equivalent success rate. Unfortunately, the significant limitations of this trial, including lack of quality cell analysis, limit the ability to draw conclusions on the effectiveness of intradiscal BMC.

## 1 Introduction

The intervertebral disc is recognized as the most common identifiable structural source of chronic low back pain (LBP) [1]. Conservative treatment options including medications, physical therapy, chiropractic treatment, and steroid injections are often ineffective. Although there is some evidence that surgical treatment for discogenic pain, typically fusion, is superior to conservative care, the results are relatively modest [2,3].

The pathoanatomy of a painful intervertebral disc has been extensively studied [4,5]. The outer portion of the disc, the annulus fibrosis, may develop tears or fissures in the collagen fibers. These fissures contain an ingrowth of vascularized granulation tissue along with extensive nerve endings. These changes are felt to contribute to the development and perpetuation of discogenic LBP [4,5].

Prior treatments aimed at disrupting the innervation to the region of the annular tear, including heat and chemical denervation, have been met with initial enthusiasm, but ultimately, disappointing results [[6], [7], [8], [9], [10], [11]]. More recently, the field of orthobiologics has attempted to address the issue. Research in areas such as stem cells, platelet-rich plasma (PRP), and specific growth factor injections has seen only limited success [[12], [13], [14], [15], [16], [17]].

Another orthobiologic agent considered a potential treatment for discogenic pain is bone marrow concentrate (BMC). BMC is obtained through a simple centrifugation of autologous bone marrow aspirate. Bone marrow aspirate/concentrate has been used extensively in orthopedic clinical settings to promote tissue healing [18,19]. Growth factors, cytokines and pluripotent stem cells within the aspirate are felt to be responsible for the ability of BMC to promote tissue healing in collagen-based structures [20,21].

BMC has been studied in animal and human trials in multiple orthopedic conditions [[20], [21], [22], [23], [24]]. Results have been promising. Bone marrow aspirate and BMC have been used extensively in spinal surgery to augment fusion with positive results [19].

A limited number of prior studies have been performed investigating the use of bone marrow concentrate for discogenic LBP [[25], [26], [27], [28], [29], [30], [31]]. At the time of this manuscript, no double-blind randomized controlled trial has been performed to evaluate the benefits of intradiscal BMC. The purpose of the current study is to determine the efficacy of a single intradiscal BMC injection for presumed discogenic LBP as well as contribute to the safety data for this novel treatment.

The preparation of BMC in this trial was through a simple concentration method of autologous bone marrow with an FDA approved Arthrex (Naples, FL) Angel flow cytometry bone marrow concentrate system. The procedure is considered to be of minimal manipulation of bone marrow tissue for homologous use as per FDA draft guidance covered under Code of Federal Regulations 361 [32,33].

## 2 Methods

The study was approved by Stirling IRB, an independent institutional review board: ID # 6075-001. The trial was registered in Clinicaltrials.gov NCT03340818. The study was performed at an outpatient interventional physiatry private practice. Participants were recruited from a group of consecutive patients from 2018 to 2022 from the investigators' (DL, SH) physiatry practice or from the partner neurosurgery practice. The study was funded by a grant from International Pain and Spine Intervention Society (formerly Spine Intervention Society). No industry funding (direct or through supplies) was provided. Participants were compensated for their time at the 3-, 6-, and 12- month visits with 50 dollars at each of the 3 visits.

### 2.1 Inclusion criteria

Age 18–55. Patients beyond 55 were not included based upon the lower prevalence of discogenic pain [34]. Numeric rating scale ≥4/10, LBP greater than leg pain. Patients met criteria indicating presumptive discogenic pain as well as requiring other potential sources of pain to be excluded. The criteria for discogenic pain included either A or B below:

A. Positive provocation discography in accordance with International Pain and Spine Society (IPSIS) guidelines [35].
B. MRI findings suggestive of discogenic LBP including either a high intensity zone region or type 1 or type 2 Modic end plate changes [[36], [37], [38]]. Along with the image findings, patients were required to undergo interventional procedures to exclude other structural sources of pain as appropriate (see below).

### 2.2 Exclusion criteria

Exclusion of non-disc structures as source of pain:

Facet joints: If the patient had non-midline pain [34], negative diagnostic medial branch blocks (in accordance with IPSIS guidelines) were required. Alternatively, patients who had previously undergone radiofrequency ablation and had less than 50 % improvement in pain were assumed to have had 2 false positive blocks and could be included (assuming A or B of inclusion criteria were met).

Sacroiliac joints: If the patient had non-midline pain below L5 or dominate buttocks pain, a negative intra-articular sacroiliac joint injection (in accordance with IPSIS guidelines) was required (<50 % improvement on NRS.)

Additional exclusion criteria: see Table 1.

**Table 1:** Exclusion criteria (in addition to exclusion of facet and sacroiliac joint mediated pain).

Exclusion Criteria
• Active moderate or severe lumbar radiculopathy
• Negative provocation discography
• Disc height less than 1/3 expected normal disc height
• Active infection
• Moderate to severe anemia, thrombocytopenia, or leukopenia
• Spinal fracture within the past 6 months
• Spondylolisthesis >3 mm
• Spondylolysis
• Severe psychological illness
• Inability to consent to procedure due to cognitive issues
• Prior surgery at the level considered to be the source of pain
• Low back surgery within the past 6 months
• Pregnant or breastfeeding females
• Severe uncontrolled medical condition
• Inflammatory arthritis
• Any cancer within the past 5 years except basal cell or squamous cell skin cancer
• Intradural disc herniation
• Coagulopathy preventing spinal injection
• Inability to stop anticoagulants other than aspirin due to other medical issues
• Exceeds 30 mg morphine equivalent per day of opioid use
• A history of alcohol or drug abuse within the past 5 years
• Use of any investigational drug within the past 30 days
• Steroid injection in the spine within the past 30 days
• Any intradiscal injection other than contrast dye or anesthetic within 6 months
• A known allergy or sensitivity to heparin or citrate
• Pending litigation involving the subject's back pain
• Central stenosis at the level to be injected with an AP diameter ≤5 mm
• Severe anaphylactic/anaphylactoid reaction to any of the medications used
• Inadequate medical insurance coverage for any subsequent tests or procedures deemed clinically
• Zygapophysial joint mediated pain
• Sacroiliac joint mediated pain

### 2.3 Power analysis

A power analysis was performed based upon the calculated success rate (>50 % improvement in pain) from Pettine et al.‘s study [26] of 61 % for the treatment arm (Our calculation of 61 % differs from Pettine et al.‘s reported higher success rate in the publication, as we included surgical patients as failures.). To estimate the success rate of a sham spinal injection procedure, the authors used the results of a level one spinal injection explanatory trial examining cultured expanded stem cells [15]. The success rate (50 % improvement on VAS) of in intradiscal injection of normal saline at 1 year was 20 % [15].

The sample size was calculated using categorical proportions, an allocation ratio of 2 (2:1 treatment vs sham), 80 % power, with a chi-squared test at a level of significance of 0.05, assuming the proportion of patients achieving success in the treatment group is 0.61(61 %) and 0.2 (20 %) in the control group. This results in the requirement of 40 patients in the treatment group and 20 patients in the control group assuming a 15 % loss to follow-up. Using an n of 40 in our proposed treatment group with 61 % success rate the 95 % CI are 44–75 %. Using an n of 20 for the sham group and 20 % success rate, the 95 % CI are 4–43 %. In this conservative scenario, the confidence intervals do not overlap.

### 2.4 Number of subjects and randomization

Initially the authors’ plan was to include 60 patients total, 40 in the treatment arm and 20 in the control arm. The randomization process for the first 47 patients was based upon a random number generator, utilized for each individual patient immediately prior to the procedure (https://www.random.org/#numbers Randomness and Integrity Services Ltd. Dublin, IR). The program was set to 2:1, treatment to sham. After 47 patients were enrolled, an interim analysis of the randomization process was performed by one of the unblinded investigators. This revealed that the computer randomization method resulted in about a 4:1 ratio, treatment to sham. In order to correct the low number of subjects in the sham group, the authors chose to proceed with a 1:1 treatment to sham randomization process for the remaining 13 patients and add 6 more patients to increase total enrollment to 66 patients (with protocol revision approval by the IRB). Randomization was then based upon random envelope assignment determined immediately prior to the injection procedure.

### 2.5 Outcome measures

Pain and function were assessed at baseline, 3, 6, and 12 months by Clinical Outcome Measurement Brief Instrument (COMBI) [39]. Numeric rating scale (NRS) and the Pain, Enjoyment, General Activity (PEG) scale are included within COMBI. Function was also assessed by Oswestry Disability Index (ODI) [40]. Primary outcome was based upon clinical success, defined as at least 50 % relief of pain from baseline to 3, 6, and 12 months. Secondary outcomes were improvement in function by ODI as well as elements of the COMBI including global impression of pain, patient specified functional outcome scale activity restoration, pain interference, medication use, other healthcare utilization, and work status [39].

Blinding: The randomization process took place immediately prior to the injection procedure. Patients were blinded to treatment assignment. The physician performing the procedure was aware of the group assignment. All patient follow-up for assessment and completion of outcome measures was performed by an investigator blinded to the treatment assignment.

To help determine the blinding success [41], immediately following the injection procedure, the patients were given a written query whether they believed they were given treatment, sham, or did not know.

### 2.6 Procedure

All procedures were performed by two of the investigators (DL and SH). Both are board certified in Physical Medicine and Rehabilitation, experienced discographers, fellowship trained in spine injections, and serve as instructors for spinal injection procedures at the national level. Both investigators have supplemental training and years of experience with bone marrow aspiration procedures.

### 2.7 Bone marrow aspiration and sham bone marrow aspiration

Pre-procedure:

The patients were offered a P.O. anxiolytic of diazepam 5–10 mg or alprazolam 0.5–2 mg to be taken 30–60 min prior to the procedure. Alternatively, patients were offered moderate sedation in the form of 2 mg–6 mg midazolam IV with the possible addition of 50–150 mcg fentanyl IV.

**Fig. 1:**
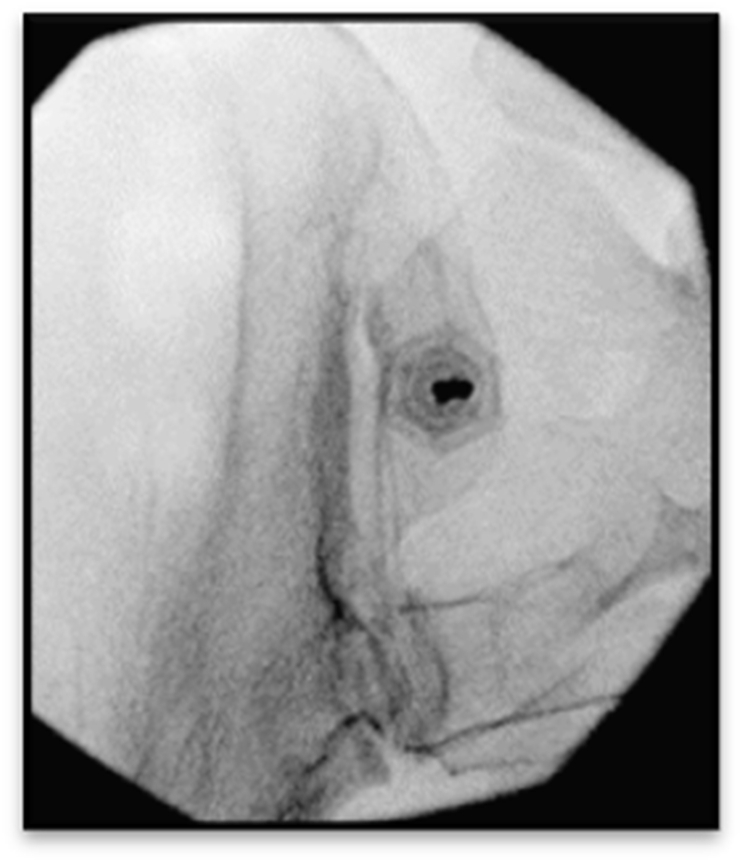
Bone marrow aspiration from the left posterior superior iliac spine.

Bone marrow aspiration procedure (Fig. 1).

The area was prepped with a chlorhexidine and alcohol solution. The drape was placed in a manner in which the procedure was not visible to the patient. The posterior superior iliac spine (PSIS) was identified by fluoroscopy. A 25-gauge needle was placed down to the periosteum, which was then anesthetized with 5 ml of ropivacaine 0.5 %. An additional 2 ml was used to anesthetize the superficial tissues and skin. The needle, syringes, and BMC filter were primed with heparin 500 units/ml. All heparin was discarded from the syringes. A total of 9 ml of anticoagulant citrate dextrose-formula (ACD-A) was placed in the syringes to prevent clotting of the aspirate and divided equally into the 6, 10 ml syringes. A 15G needle with a stylet was directed manually down to the periosteum under intermittent fluoroscopic guidance. The Arrow Oncontrol (Teleflex, Morrisville, NC) drill aspiration system was used with a 15G aspiration needle. The drill, within the sterile cover, was then placed and “locked” onto the aspiration needle. The aspiration needle was then advanced by the drill into the cavity of the ilium. The stylet was then removed and the syringe attached. A total of 51 ml of bone marrow aspirate was slowly withdrawn by negative pressure from 3 sites via 10 ml syringes (each 10 ml syringe also contained 1.5 ml ACD-A anticoagulant). At each of the 3 insertion sites the needle was rotated 90° with each 5 ml of aspiration to localize different areas of aspirate with approximately 30 s aspiration time for each 5 ml volume. A single side aspiration was performed if one to two discs were to be injected. For greater than two discs, the above procedure was completed twice using both PSIS in which a total of 102 ml of bone marrow was withdrawn (combined with 18 ml ACD-A anticoagulant).

The aspirate was then placed through a 150-μm bone marrow filter to remove any calcific fragments. Using the Arthrex (Naples, FL) Angel processing system, the bone marrow was concentrated by means of flow cytometry in a sterile closed system. The 7 % hematocrit setting was used as directed by the manufacturer for optimal stem cell concentration. Strict sterile technique was maintained during processing and transferring of the BMC following the processing.

Lab analysis: For five of the patients in the active treatment group, one to two ml of bone marrow aspirate (BMA), as well as one to two ml of BMC, were sent to an independent lab for analysis. Analysis entailed nucleated cell count, cell viability, CD34^+^ concentration, and count of fibroblast colony forming units. The sample choices were non-random and based upon a single disc to be injected as only 2–3 ml of the 5 ml of BMC concentrate produced would be used. Fresh samples of both the BMA and BMC were sent from Virginia Beach, VA to an independent lab, Biofyl, in Ft Meyers, Florida as instructed by Biofyl.

### 2.8 Sham bone marrow aspiration

The patient was draped in a manner in which the procedure was not visible. The above setup was identical to the actual bone marrow aspiration. The skin and superficial tissue was anesthetized in a similar manner. The needle was not attached to the drill, but the drill trigger was squeezed so the patient heard the noise of the drill. Simultaneously, a 21G needle was tapped against the periosteum. To simulate the pressure of aspirating the bone marrow, 3 ml of normal saline was injected just superficial to the periosteum. The length of time of the sham procedure was similar to the actual bone marrow aspirate procedure.

### 2.9 Intradiscal BMC injection procedure

Levels to be injected were determined by A or B below.

A: If discography was performed: Disc level(s) determined to be positive on prior discography performed within the preceding 6 months using International Pain and Spine Intervention Society standards [35].

B: As discography was not required in the inclusion criteria, disc levels were determined by clinical and image findings. MRI findings of high intensity zone, Modic changes type 1 or 2, and decreased T2 disc signal were used [[36], [37], [38],^42^] in conjunction with the general location of pain and tenderness on exam (upper, middle, or lower lumbar location).

**Fig. 2:**
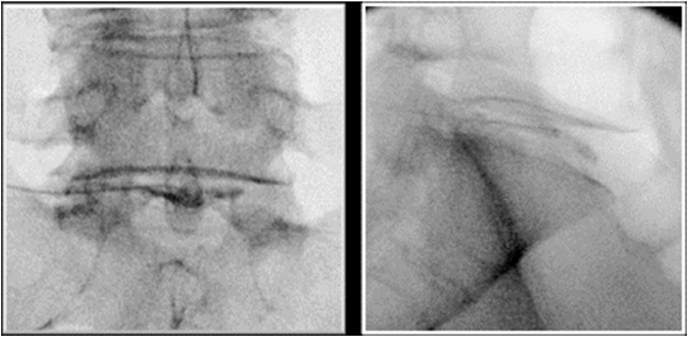
AP and lateral intradiscal injection at L5/S1.

Active treatment procedure (Fig. 2).

The patient was placed in a prone position in an outpatient fluoroscopy suite. Strict sterile technique was observed. The lower back was cleansed with chlorhexidine mixed with alcohol and covered with a sterile drape. The patient was draped in a manner in which the procedure was not visible. A standard posterolateral extrapedicular discogram technique was used under intermittent fluoroscopic imaging for each level previously determined to be a presumptive pain generator. The skin and superficial tissues were anesthetized with 2–5 ml of lidocaine 1 %. The needle tip of a 22-gauge or 25-gauge needle with stylet, single styletted needle technique, was directed toward the disc into Kambin's triangle. Depending upon the patient's tolerance of the procedure, one ml ropivacaine 0.5 % may have been injected just outside the disc annulus into the epidural space (infraneural transforaminal epidural technique). The stylet was then replaced and directed into the disc nucleus. A volume of 0.6 ml of contrast solution consisting of 0.4 ml Omnipaque 240 or 300 contrast agent with 0.2 ml of cefazolin 330 mg/ml (for a total of 66 mg, at a final concentration of 18 mg/ml based upon 3.5 ml total volume) (or in cefazolin allergic patients 0.4 ml of 40 mg/ml gentamicin) was injected through small bore tubing under live fluoroscopy to confirm intranuclear location. Two to three ml of previously prepared autologous BMC was then injected into the disc. Thus, a total volume of 2.6–3.6 ml was injected into each of the presumed pain-generating discs during the treatment. There were instances when the disc could not accommodate the full volume of BMC due to high pressure (based on manual pressure estimation). At least 1.5 ml of BMC needed to be injected into a disc to be considered a completed treatment. If the patient was experiencing severe discomfort during the BMC injection, the physician added 0.2–0.5 ml of ropivacaine 0.2 %–1 % into the disc. Immediately after the solution was injected, the needle was removed.

### 2.10 Sham treatment procedure

As it was not felt ethical to puncture a disc for a placebo treatment [42], it was elected to simulate the intradiscal injection with a 22G needle tapping at the corresponding transverse process as well as a rapid intramuscular injection of 3 ml of contrast agent on the dorsal surface of the corresponding transverse process cephalad to the disc level. For example, if the L5/S1 disc was the “treatment” disc, the corresponding sham was tapping on the L5 transverse process and rapid injection of 3 ml of contrast agent. The length of the sham procedure was prolonged to mirror the active treatment procedure.

The patient was placed in a prone position in an outpatient fluoroscopy suite. Strict sterile technique was observed. The lower back was cleansed with chlorhexidine mixed with alcohol and covered with a sterile drape. The patient was draped in a manner that the injection was not visible. The skin was anesthetized with 2 ml lidocaine 1 % at each level to be injected. A 22G spinal needle was advanced to the transverse process at the superior level of each disc to be “treated”. The needle contacted the periosteum and a tapping maneuver was performed. With the needle tip remaining on the periosteum, three ml of contrast agent, Omnipaque 240, was rapidly injected just dorsal to the transverse process.

### 2.11 Post procedure

Patients completed the blinding assessment query as to whether they believed they received the active treatment, sham, or they did not know. They were offered a short course (ten tablets) of post-procedure pain medication such as hydrocodone/acetaminophen 5/325 mg or oxycodone/acetaminophen 5/325 mg to take 1–2 tabs q 4-6hrs prn post-procedure pain. They were instructed not to engage in any strenuous activity for two weeks after the treatment.

The validity of the sham procedure was performed through a questionnaire following the procedure [43]. This was provided after the intradiscal injection of the BMC or following the sham intradiscal injection. Patients checked one of 3 boxes to indicate whether they believed they received the actual BMC injection, the sham injection, or were unsure.

### 2.12 Co-interventions

All patients continued any home exercise program. No specific physical therapy or other interventions were prescribed. Patients were instructed to avoid NSAIDS for 6 weeks but could continue other medications previously prescribed.

### 2.13 Analysis

Demographic and baseline characteristics as well as rates of success (≥50 % improvement in NRS, ≥30 % improvement in NRS, ≥30 % improvement in ODI, and much/very much improved Global Impression of Change) between the treatment and sham cohorts were analyzed using Mann-Whitney tests and chi-square tests.

Mann-Whitney tests, chi-square tests, and Fisher's exact tests were used to analyze differences in age, BMI, pain duration, Modic changes, high intensity zones (HIZ), episodic low back pain, and opioid use between patients with successful (≥50 % improvement in NRS) and unsuccessful (<50 % improvement in NRS) treatment. Subsequent multivariable logistic regression models were run within the treatment cohort to evaluate predictors of success, reducing the model in a stepwise manner as needed to prevent overfitting. Analyses were run in RStudio using a two-sided level of significance of 0.05.

## 3 Results

**Fig. 3:**
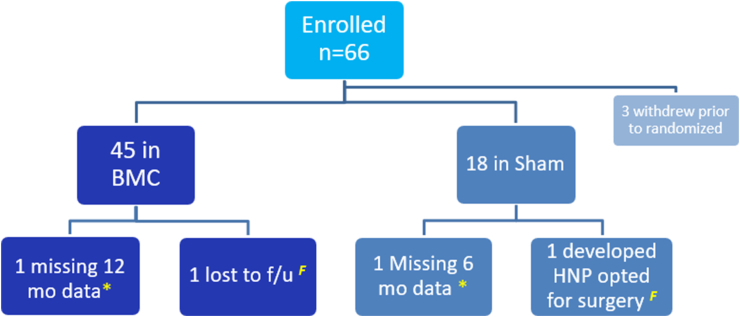
Flow diagram of patient assignment and follow up. ∗ Treated in analysis with last data carried forward. ^*F*^ Treated in analysis as failures.

A total of 63 patients participated in the trial (Fig. 3). Forty-five patients underwent the treatment with intradiscal BMC, and 18 patients underwent the sham procedure. The majority of patients underwent the procedure at a single level. There was no significant difference in the number of treated discs in the BMC group and the number of index discs in the sham group (Table 2).

**Table 2:** Number of disc levels injected.

Number of Levels	Discs Injected in BMC Group N (%)	Index Discs in Sham Group N (%)	p-value
1 level	22 (49 %)	4 (22 %)	
2 levels	18 (40 %)	11 (61 %)	p = 0.155
3 levels	5 (11 %)	3 (17 %)	
Total	45	18	

The mean patient age was 41.7 (range = 22–55) years and the mean BMI was 28.5 (range = 17.9–48.0). On average, patients experienced pain for 9.5 (range = 1–35) years and, at baseline, reported an average NRS pain score of 5.6 (range = 4–9) out of 10. Demographic and baseline characteristics did not significantly differ between treatment groups (Table 3).

**Table 3:** Baseline demographics and characteristics. Data is presented as means ± standard deviations for continuous variables, and n (%) for categorical variables.

Baseline Demographics & Characteristics	Overall	BMC	Sham	
	(N = 63)	(N = 45)	(N = 18)	p-value
Age (Years)	41.7 (8.5)	41.8 (8.8)	41.5 (8.0)	0.784
BMI	28.5 (6.3)	28.5 (7.1)	28.5 (3.9)	0.514
Modic Changes				
Negative	36 (57.1 %)	25 (55.6 %)	11 (61.1 %)	0.904
Positive	27 (42.9 %)	20 (44.4 %)	7 (38.9 %)	
HIZ				
Negative	17 (27.0 %)	11 (24.4 %)	6 (33.3 %)	0.686
Positive	46 (73.0 %)	34 (75.6 %)	12 (66.7 %)	
Episodic low back pain				
No	25 (39.7 %)	19 (42.2 %)	6 (33.3 %)	0.714
Yes	38 (60.3 %)	26 (57.8 %)	12 (66.7 %)	
Duration of Pain (years)	9.5 (7.1)	10.1 (7.2)	8.1 (6.9)	0.238
Baseline NRS	5.6 (1.5)	5.7 (1.4)	5.5 (1.7)	0.402
Baseline ODI	31.9 (11.1)	33.4 (11.2)	28.3 (10.1)	0.170
Baseline PEG	5.8 (1.5)	5.9 (1.4)	5.7 (1.7)	0.452

Modic = Modic endplate changes type 1 or 2, HIZ = High intensity zone on MRI. NRS= Numeric rating scale, ODI = Oswestry disability index, PEG = Pain, enjoyment of life, and general activity scale.

## 4 Adverse events

There were no cases of discitis. One patient in the treatment group had severe pain beginning a few hours following the procedure which was relieved with oxycodone and the pain decreased significantly by day 2. One patient in the sham group developed a large disc herniation and opted for surgical intervention. This was deemed by the lead investigator to be unrelated to the sham procedure, as the needle did not penetrate the disc during the mock disc injection.

The sham procedure was considered valid based upon the post-procedure questionnaire. Approximately 50 % of participants in both the treatment and the sham group correctly presumed their actual group assignment [41,43]. There was no significant difference between the group assumption of assignment (Table 4).

### 4.1 Bivariate analysis

For the primary outcome (>50 % improvement in NRS), 40 % [95 % CI: 27–50 %] of patients in the BMC group achieved success at 3 and 6 months, and 44 % [95 % CI: 31–59 %] achieved success at 12 months. These results were statistically indistinguishable from the sham group which achieved success rates of 33 % [95 % CI: 15–56 %], 39 % [95 % CI: 20–61 %], and 56 % [95 % CI: 34–75 %] at 3, 6, and 12 months, respectively. The proportion of patients who achieved >30 % improvement in NRS were also statistically similar between groups. The improvement in disability as measured by the ODI, and the proportion of patients who were much improved or very much improved on the global perception of pain, both showed no significant differences between groups (Table 5). A box plot graph for percent change in NRS and ODI also demonstrates lack of difference between groups (Table 6, Table 7).

**Table 4:** Validity of the sham procedure.

Patient Perception	BMC (N = 45)	Sham (N = 18)	
	N (%)	N (%)	p-value
Believed Received BMC	22 (49 %)	5 (28 %)	0.194
Believed Received Sham	19 (42 %)	9 (50 %)	
Unsure	4 (9 %)	4 (22 %)

**Table 5:** Improvement in pain, function and Global Impression of Change. NRS = Numeric rating scale, ODI = Oswestry disability scale, PGIC = Patient Global Impression of Change.

	BMC (N = 45)		Sham (N = 18)		
	N	% [95 % CI]	N	% [95 % CI]	p-value
>50 % Reduction in NRS					
3 Months	18	40.0 % [27–50 %]	6	33.3 % [16–56 %]	0.838
6 Months	18	40.0 % [27–50 %]	7	38.9 % [20–61 %]	> 0.999
12 Months	20	44.4 % [31–59 %]	10	55.6 % [34–75 %]	0.604
>30 % Reduction in NRS					
3 Months	23	51.1 % [37–65 %]	6	33.3 % [16–56 %]	0.318
6 Months	26	57.8 % [43–71 %]	8	44.4 % [25–66 %]	0.497
12 Months	27	60.0 % [45–73 %]	10	55.6 % [34–75 %]	0.968
>30 % Reduction in ODI					
3 Months	23	51.1 % [37–65 %]	6	33.3 % [16–56 %]	0.318
6 Months	24	53.3 % [39–67 %]	8	44.4 % [25–66 %]	0.720
12 Months	25	55.6 % [41–69 %]	11	61.1 % [39–80 %]	0.904
PGIC Much or very much improved					
3 Months	15	33.3 % [21–48 %]	4	22.2 % [9–45 %]	0.573
6 Months	19	42.2 % [29–57 %]	6	33.3 % [16–56 %]	0.714
12 Months	21	46.7 % [33–61 %]	6	33.3 % [16–56 %]	0.494

**Table 6:**
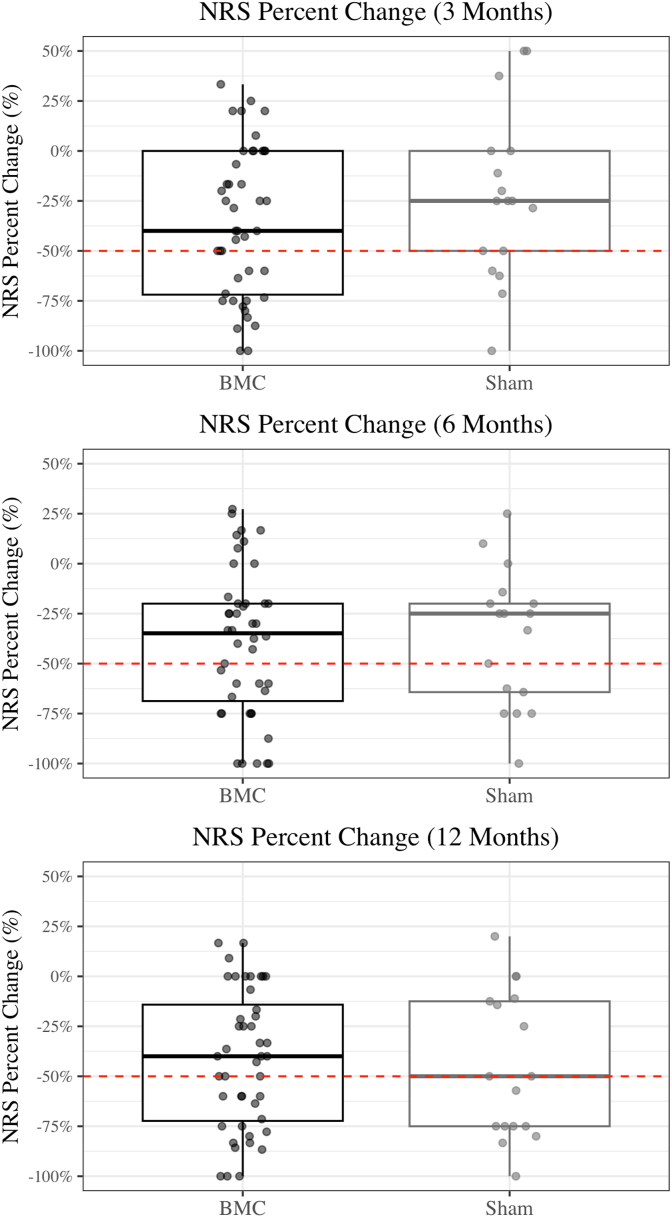
Box plot for percent change in NRS for BMC and Sham at 3-, 6- and 12-month follow-up. NRS = Numeric rating scale.

**Table 7:**
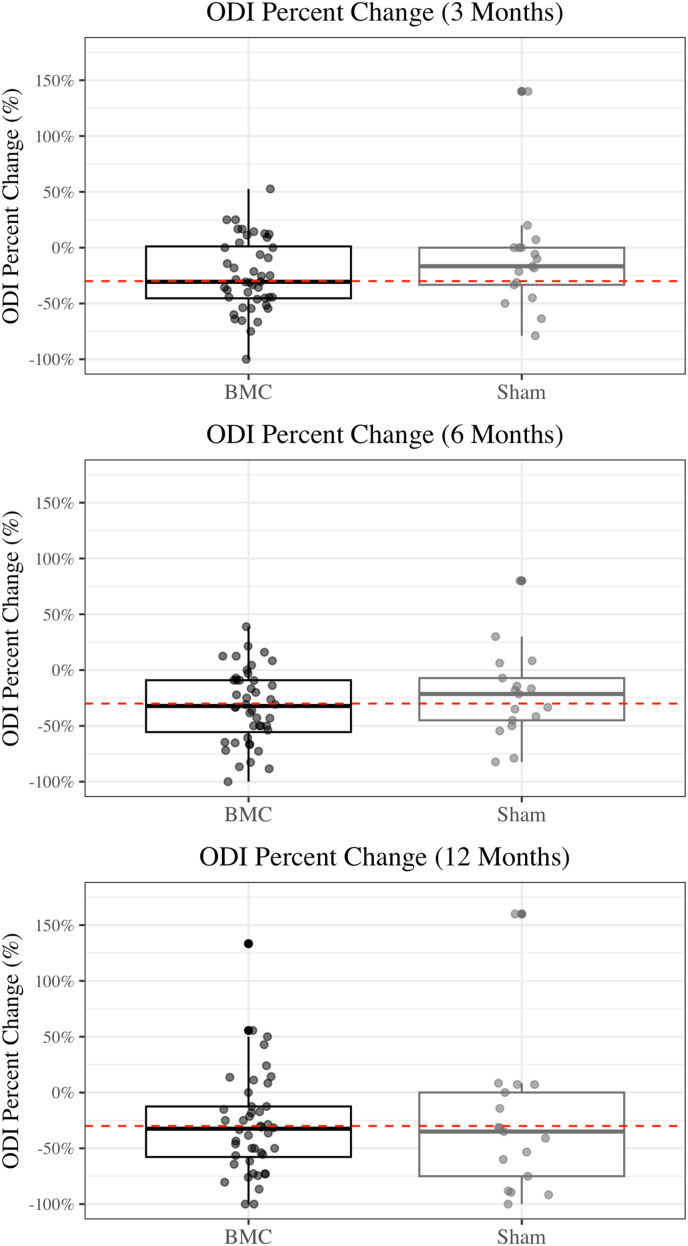
Box plot for percent change in ODI for BMC and Sham at 3-, 6- and 12-month follow-up. ODI = Oswestry disability index.

Patient demographic and clinical variables were not significantly associated with successful outcome in the entire subject population (≥50 % improvement in NRS) at 3 and 6 months, but lower BMI was associated with success at 12 months (p = 0.033) (Table 8).

**Table 8:** Demographic and clinical variables vs. successful outcome (>50 % improvement in Numeric rating scale). Modic = Modic endplate changes type 1 or 2, HIZ = High intensity zone on MRI, Episodic = history of episodic low back pain, Opioid partial included/excluded refers to tramadol.

	3 Months					6 Months					12 Months				
	Not Successful (N = 39)		Successful (N = 24)			Not Successful (N = 38)		Successful (N = 25)			Not Successful (N = 33)		Successful (N = 30)		
	Median	Range	Median	Range	p-value	Median	Range	Median	Range	p-value	Median	Range	Median	Range	p-value
Duration	8	1–35	10	1–23	0.780	6.5	1–25	9.5	1–35	0.295	7.5	1–20	8.5	1–35	0.921
Age	43	22–55	40.5	25–55	0.605	42.5	22–55	42	25–55	0.518	43	26–55	40.5	22–55	0.577
BMI	27	17.9–48	26.9	18–36	0.623	26.7	17.9–48	29	18–44	0.795	28.8	22–48	26	17.9–36	0.033∗
	N	%	N	%	p-value	N	%	N	%	p-value	N	%	N	%	p-value
Modic Changes															
Negative	22	56.4 %	14	58.3 %	>0.999	21	55.3 %	15	60.0 %	0.911	18	54.5 %	18	60.0 %	0.856
Positive	17	43.6 %	10	41.7 %		17	44.7 %	10	40.0 %		15	45.5 %	12	40.0 %	
HIZ															
Negative	9	23.1 %	8	33.3 %	0.550	11	28.9 %	6	24.0 %	0.887	8	24.2 %	9	30.0 %	0.818
Positive	30	76.9 %	16	66.7 %		27	71.1 %	19	76.0 %		25	75.8 %	21	70.0 %	
Episodic															
No	15	38.5 %	10	41.7 %	>0.999	15	39.5 %	10	40.0 %	>0.999	14	42.4 %	11	36.7 %	0.835
Yes	24	61.5 %	14	58.3 %		23	60.5 %	15	60.0 %		19	57.6 %	19	63.3 %	
Opioid/Narcotic (Partial Included)															
No	33	84.6 %	20	83.3 %	>0.999	32	84.2 %	21	84.0 %	>0.999	28	84.8 %	25	83.3 %	>0.999
Yes	6	15.4 %	4	16.7 %		6	15.8 %	4	16.0 %		5	15.2 %	5	16.7 %	
Opioid/Narcotic (Partial Excluded)															
No	36	92.3 %	21	87.5 %	0.850	34	89.5 %	23	92.0 %	>0.999	30	90.9 %	27	90.0 %	>0.999
Yes	3	7.7 %	3	12.5 %		4	10.5 %	2	8.0 %		3	9.1 %	3	10.0 %	

### 4.2 Multivariable analysis

When looking at a sub-analysis of the BMC treatment group, higher BMI was found to be associated with a decreased odds of successful at 12 months (≥50 % improvement in NRS) after adjusting for age, Modic changes, and presence of an HIZ (odds ratio = 0.85, 95 % CI = [0.72, 0.96], p = 0.023. No significant associations were found when looking at 3- or 6-month success (Table 9). There was no significant association with duration of pain and success of the treatment at any time point. No significant difference was seen in analysis based upon number of discs injected (Table 10).

**Table 9:** Multivariable logistic regression models analyzing factors associated with ≥50 % improvement in Numeric Rating Scale in the treatment group. Modic = Modic endplate changes type 1 or 2, HIZ = High intensity zone on MRI. ∗ Denotes statistical significance.

Time Point	Variable	Odds Ratio [95 % CI]	p-value
3 Months	Age	0.98 [0.91, 1.05]	0.547
	BMI	0.95 [0.85, 1.04]	0.290
	Modic +	0.97 [0.26, 3.63]	0.961
	HIZ +	0.85 [0.16, 5.05]	0.854
6 Months	Age	1.03 [0.96, 1.11]	0.467
	BMI	1.01 [0.92, 1.11]	0.842
	Modic +	0.93 [0.25, 3.44]	0.916
	HIZ +	2.17 [0.4, 16.99]	0.398
12 Months	Age	0.98 [0.90, 1.05]	0.528
	BMI	0.85 [0.72, 0.96]	0.023∗
	Modic +	1.08 [0.26, 4.61]	0.919
	HIZ +	0.39 [0.06, 2.41]	0.320

**Table 10:** Sub-analysis of pain relief by NRS, functional improvement by ODI, patient perception of change, as well as validity of sham based upon the number of discs injected. NRS = Numeric rating scale, ODI = Oswestry disability index, GIC = Global Impression of Change.

	BMC: 1 Disc (N = 22)		BMC: 2 Discs (N = 18)		BMC: 3 Discs (N = 5)		Placebo (N = 18)		
	N	%					N	%	p-value
≥50 % Reduction in NRS									
3 Months	9	40.9 %	7	38.9 %	2	40.0 %	6	33.3 %	0.838
6 Months	10	45.5 %	6	33.3 %	2	40.0 %	7	38.9 %	>0.999
12 Months	8	36.4 %	10	55.6 %	2	40.0 %	10	55.6 %	0.604
>30 % Reduction in NRS									
3 Months	11	50.0 %	8	44.4 %	4	80.0 %	6	33.3 %	0.318
6 Months	13	59.1 %	10	55.6 %	3	60.0 %	8	44.4 %	0.497
12 Months	13	59.1 %	10	55.6 %	4	80.0 %	10	55.6 %	0.968
>30 % Reduction in ODI									
3 Months	9	40.9 %	11	61.1 %	3	60.0 %	6	33.3 %	0.318
6 Months	11	50.0 %	10	55.6 %	3	60.0 %	8	44.4 %	0.720
12 Months	10	45.5 %	12	66.7 %	3	60.0 %	11	61.1 %	0.904
GIC (Much or Very Much Improved)									
3 Months	8	38.1 %	6	33.3 %	1	20.0 %	4	22.2 %	0.573
6 Months	10	47.6 %	6	33.3 %	3	60.0 %	6	33.3 %	0.714
12 Months	11	52.4 %	8	47.1 %	2	40.0 %	6	33.3 %	0.494
Patient Perception									
Believed Received BMC	8	36.4 %	12	66.7 %	2	40.0 %	5	27.8 %	0.194
Believed Received Sham	2	9.1 %	1	5.6 %	1	20.0 %	9	50.0 %	
Unsure	12	54.5 %	5	27.8 %	2	40.0 %	4	22.2 %	

### 4.3 Lab analysis

Lab analysis for the 5 non-random samples of bone marrow aspiration and BMC were overall non diagnostic. Fresh samples of both the BMA and BMC were sent from Virginia Beach, VA to an independent lab, Biofyl, in Ft Meyers, Florida as instructed by Biofyl. Unfortunately, per communication with Biofyl, analysis was extremely limited by the condition of the samples in which the analysis was performed 1–6 days after collection. No living cells were present and only cell count analysis was possible (Table 11). The accuracy of the cell count analysis is questionable. The 5 samples were performed only for injectate analysis and were inadequate in number and accuracy to perform any further analysis with respect to correlation to success rate.

**Table 11:** Bone marrow aspirate and concentrate sample analysis (accuracy limited by condition of sample upon arrival at distant facility).

	Bone Marrow Aspirate	Bone Marrow Concentrate
Mean number of nucleated cells x 106 per ml (range)	15 (10–14)	35 (16–53)
Mean number of platelets x 106 per ml (range)	132 (39–262)	558 (93–1362)

## 5 Discussion

This is the first double-blind sham-controlled trial of intradiscal BMC for discogenic LBP. Although 40 % of the treatment group obtained at least 50 % improvement in pain at 3 and 6 months follow up, this was statistically equivalent to the sham group (33 % and 39 %). One-year outcomes demonstrated similar findings, with 44 % in the BMC group with at least 50 % relief vs 56 % in the sham group. The large improvement in function as measured by ODI in the BMC group was also mirrored in the sham group. Despite the magnitude of improvement in pain and function seen over baseline pain and function with intradiscal BMC, there was no statistical significance over the sham procedure.

Prior studies have examined the use of bone marrow concentrate for discogenic low back pain [[25], [26], [27], [28], [29], [30], [31]]. Two prospective trials have been performed with encouraging results [[25], [26], [27], [28], [29], [30]]. In Pettine et al.‘s trial, 26 patients underwent intradiscal BMC for presumed discogenic LBP [26]. The results of this small trial were very encouraging including an average decrease in pain of 71 % at 2 years for the 21 patients who avoided surgery.

As the authors provided raw data in the publication, categorical outcome for 50 % improvement in pain, although not explicitly stated, was calculated to be 61 % (95 %CI 43–77 %) at one year. This was statistically equivalent to the one-year outcome in the current trial of 44 % (95 % CI 29–59 %). Haines et al. also performed a prospective observational trial investigation intradiscal BMC (30z). Thirty-two patients underwent the treatment for presumed intradiscal pain. Mean data were provided with NRS scores improving from 5.4 at baseline to 3.2 at 1 year.

Recently, there was a publication of a RCT of 40 patients receiving either intradiscal PRP or intradiscal BMC compared to a control group receiving a trigger point injection. The authors reported equivalent success of PRP and BMC with statically significant improvement over the trigger point control group [31]. Unfortunately, this was open label and therefore was subject to inherent bias in comparing results of the treatment groups to that of the control group.

As with all investigational therapies, evaluation of safety is always of primary importance. There were no significant complications from this trial which included 45 patients undergoing intradiscal BMC. Discitis is clearly of concern for any intradiscal procedure. In the current trial, we did include intradiscal antibiotic with the BMC for discitis prophylaxis. There is controversy regarding the use of prophylactic IV or intradiscal antibiotic [44]. Although there were no cases of discitis in this trial or any of the other intradiscal BMC published trials [[25], [26], [27], [28], [29], [30], [31]], there have been 3 case reports of discitis following intradiscal BMC described by Jerome and colleagues [45]. One was likely an aseptic chemical discitis which failed to improve with IV antibiotic but resolved with dexamethasone. Two others were clearly bacterial. In both cases, prophylactic IV antibiotics were used, and one case included 500ug intradiscal gentamycin with a total volume of 2.5 ml injected into the disc. It should be noted that the dosage in that case was much less than the recommended minimal intradiscal gentamycin dosage of 1 mg/ml [46,47]. Although there are possible deleterious effects of intradiscal antibiotic on disc cells [48], prophylactic IV antibiotic for discitis is extremely difficult to time appropriately [46] and disc penetration is not consistent [49]. Regardless, it should be recognized that placing antibiotic into the disc, may have adversely affected the outcome of our treatment.

This trial has several other limitations. The authors chose to limit patient age to 55 and younger. This was based upon the study by Depalma et al. [34] that showed that at age 55 and less, the predicted probability of a discogenic source of pain is higher than any other source. Positive discography was not required for inclusion. It was felt that the potential risk of discography [50] would outweigh the potential risk of injecting a seemingly benign autologous substance into a nonpainful disc. It was also assumed that performing the BMC injection at the time of discography could not be appropriately performed as the disc volume capacity would be inadequate to inject the BMC should the discography be performed by IPSIS guidelines [35].

Another limitation of this study is the use of a non-intradiscal sham procedure rather than an intradiscal placebo. This was based upon the adverse effects of needle injury to the disc [42]. Of note, we did perform a post procedure questionnaire to assess the patients’ belief of receiving the treatment or sham [41,43]. This demonstrated no significant difference in patient belief of treatment assignment.

The most profound limitation of this trial, however, is the lack of internal control of the BMC injectate quality. Unfortunately, our limited analysis of only five samples was deficient. Ideally every BMC would have a quality lab analysis sample to determine the biologic properties, in particular the number of mesenchymal stem cells by means of colony forming units. Our limited samples were in suboptimal condition upon arriving at the distant lab leaving the results of analysis questionable, and, as no living cells were present, appropriate culture analysis was not feasible.

Conclusion: Although this double-blind sham-controlled trial showed meaningful improvement in pain and function of a single intradiscal injection of bone marrow concentrate, it failed to demonstrate significance over a sham procedure. Unfortunately, the significant limitations of this trial, including lack of quality cell analysis, limit the ability to draw conclusions on the effectiveness of intradiscal BMC. Further research is needed to help determine the efficacy of this treatment.
